# Animal Models of Cancer-Associated Hypercalcemia

**DOI:** 10.3390/vetsci4020021

**Published:** 2017-04-13

**Authors:** Nicole A. Kohart, Said M. Elshafae, Justin T. Breitbach, Thomas J. Rosol

**Affiliations:** 1 Department of Veterinary Biosciences, College of Veterinary Medicine, The Ohio State University, Columbus, OH 43210, USA; kohart.6@osu.edu (N.A.K.); Elshafae.1@osu.edu (S.M.E.); breitbach.1@osu.edu (J.T.B.); 2 Department of Pathology, Faculty of Veterinary Medicine, Benha University, Banha 13511, Egypt

**Keywords:** hypercalcemia, paraneoplastic syndrome, cat, comparative oncology, dog, HHM, PTHrP, bone metastasis

## Abstract

Cancer-associated hypercalcemia (CAH) is a frequently-occurring paraneoplastic syndrome that contributes to substantial patient morbidity and occurs in both humans and animals. Patients with CAH are often characterized by markedly elevated serum calcium concentrations that result in a range of clinical symptoms involving the nervous, gastrointestinal and urinary systems. CAH is caused by two principle mechanisms; humorally-mediated and/or through local osteolytic bone metastasis resulting in excessive calcium release from resorbed bone. Humoral hypercalcemia of malignancy (HHM) is the most common mechanism and is due to the production and release of tumor-associated cytokines and humoral factors, such as parathyroid hormone-related protein (PTHrP), that act at distant sites to increase serum calcium concentrations. Local osteolytic hypercalcemia (LOH) occurs when primary or metastatic bone tumors act locally by releasing factors that stimulate osteoclast activity and bone resorption. LOH is a less frequent cause of CAH and in some cases can induce hypercalcemia in concert with HHM. Rarely, ectopic production of parathyroid hormone has been described. PTHrP-mediated hypercalcemia is the most common mechanism of CAH in human and canine malignancies and is recognized in other domestic species. Spontaneous and experimentally-induced animal models have been developed to study the mechanisms of CAH. These models have been essential for the evaluation of novel approaches and adjuvant therapies to manage CAH. This review will highlight the comparative aspects of CAH in humans and animals with a discussion of the available animal models used to study the pathogenesis of this important clinical syndrome.

## 1. Introduction

Cancer-associated hypercalcemia (CAH) is associated with a wide variety of cancers in both humans and animals (Table 1). CAH is often associated with disseminated cancers, and 80% of affected human patients die within a year of diagnosis [1]. The incidence is reported to be between 20% and 30% in human patients with cancer. In humans, CAH occurs with hematopoietic malignancies, such as adult T-cell leukemia/lymphoma, diffuse B cell lymphoma and multiple myeloma, as well as carcinomas of the lung, kidney, breast, larynx, esophagus, cervix and vulva and occasionally in prostate cancer, colorectal cancers and neuroendocrine malignancies [2,3,4,5,6]. Greater than 50% of all CAH cases are associated with lung cancer, breast cancer and multiple myeloma [7]. Classic biochemical abnormalities include hypercalcemia, hypophosphatemia, hypercalciuria (often with decreased fractional calcium excretion), increased fractional excretion of phosphorous and increased nephrogenous cyclic adenosine monophosphate (cAMP) [8,9]. Serum 1,25-dihydroxyvitamin D (calcitriol) concentrations are usually low, but can be normal or high depending on the etiology of the hypercalcemia [10,11]. Patients with high serum ionized calcium concentrations present with symptoms affecting various body organs, including the gastrointestinal, nervous or urinary systems. Ionized serum calcium (Ca^2+^) is the biologically-active form of calcium and should be measured when the test is available since it is more informative than total serum calcium concentration, which also includes the protein bound and complexed forms of serum calcium. The severity of disease often correlates with the magnitude of hypercalcemia, the rapidity of increase in calcium and the overall medical condition of the patient [12]. Early signs include anorexia, vomiting, nausea, lethargy, fatigue, polyuria and polydipsia. As serum calcium levels rise, patients can progress to renal failure, cardiac arrhythmias, coma and ultimately death [2,4]. 

**Figure 1:**
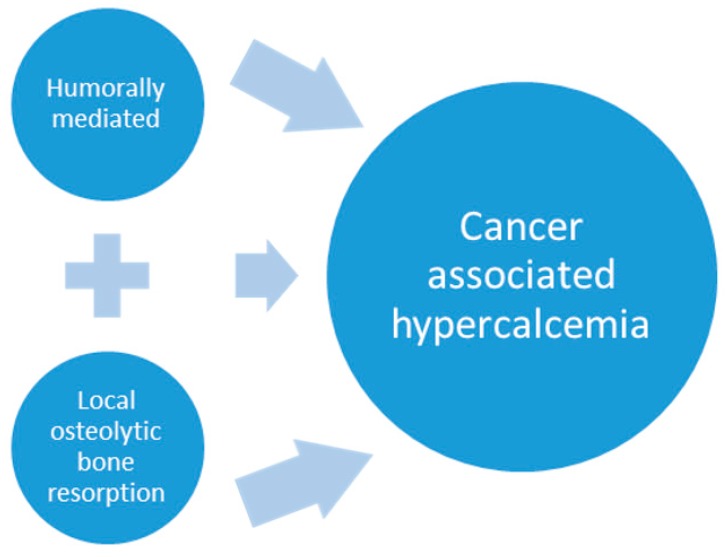
Cancer-associated hypercalcemia is mediated by two principle mechanisms: humoral hypercalcemia of malignancy with the production and release of tumor-associated hormones and/or cytokines that disrupt calcium homeostasis at the level of the bone, kidney and intestine and pathologic bone resorption from metastatic and primary bone tumors that produce factors that act locally to promote osteoclast differentiation and activity. Similar tumor-produced factors play a role in both mechanisms. Metastatic bone lesions result in focal pathologic bone resorption and may also increase serum calcium by humoral mechanisms.

Cancer-associated hypercalcemia is mediated by two principal mechanisms that include: humoral hypercalcemia of malignancy (HHM) due to circulating tumor-produced factors and hypercalcemia resulting from primary bone tumors or metastases with local osteolytic bone resorption (LOH) (concepts are illustrated in Figure 1 below). Additional mechanisms have been described, although they are rare. These include ectopic parathyroid hormone (PTH) production or primary hyperparathyroidism that coexists with malignancy [4,8,46,47]. This review will focus on HHM; however, it is worth mentioning that metastatic bone tumors release factors that can function to induce both local osteolytic bone resorption and systemic HHM.

### 1.1. Factors Associated with Humoral Hypercalcemia of Malignancy

There are several tumor-produced hormones, factors and cytokines that are capable of acting at distant sites to promote widespread pathologic bone resorption and renal reabsorption of calcium. These factors may work solely or synergistically to act on bone cells or renal tubular epithelium. The discussion in this report focuses on factors that are known to play a significant role in paraneoplastic hypercalcemia. 

Parathyroid hormone-related protein (PTHrP): Humoral hypercalcemia of malignancy accounts for over 80% of CAH in human patients [48]. HHM is caused by secretion of factors secreted by cancer cells that disrupt calcium homeostasis in the bone, kidney and intestine. Circulating parathyroid hormone-related protein (PTHrP) has been shown to be a central player in the pathogenesis of HHM. PTHrP is a polyhormone with alternative mRNA splicing and post-translational modifications that functions in an endocrine, paracrine, autocrine and intracrine fashion. Since the discovery of PTHrP in 1987, it has been appreciated that PTHrP has diverse roles in normal physiologic functions, including the regulation of cartilage differentiation, bone formation, bone resorption, tooth eruption, mammary gland differentiation and function, the immune system, fetal calcium balance, epithelial-stromal interactions and multiple other actions [49,50,51]. PTHrP has a notable role in cancer to promote tumor cell proliferation, survival, invasion and mediate hypercalcemia [52,53]. Increased circulating plasma concentrations of PTHrP occur in patients with CAH with and without evidence of bone metastasis [12,46,54,55,56,57]. The endocrine and paracrine role of PTHrP in CAH has been well defined through the use of animal models of HHM and clinical investigations in veterinary and human medicine. Eight of the first thirteen amino acids of PTHrP are homologous to parathyroid hormone (PTH), a hormone that regulates serum calcium concentration. PTHrP induces hypercalcemia in a parathyroid hormone (PTH)-like manner by binding to and activating the common PTH/PTHrP (PTH1) receptor at the surface of renal tubular epithelial cells and osteoblasts. PTHrP enhances renal reabsorption of calcium in the ascending loop of Henle and distal convoluted tubule. In bone, PTHrP binds to the PTH1 receptor on osteoblasts, osteocytes and bone stromal cells and activates signal transduction through the cyclic adenosine monophosphate/protein kinase A (cAMP/PKA) cascade [58]. This pathway upregulates the expression of the receptor activator for NF-kB ligand (RANKL) in osteoblasts. When RANKL binds to the RANK receptor on osteoclasts, the NFAT, JNK and p38 pathways are induced and stimulate osteoclast differentiation, survival and bone resorbing activity [59]. Other factors secreted by cancer cells in patients with HHM may have a synergistic and/or additive effect with PTHrP. Such factors include osteoclast stimulatory factors, such as interleukin 1 (IL-1), IL-6, tumor necrosis factor-α (TNF-α), granulocyte-macrophage colony stimulating factor (GM-CSF), macrophage colony stimulating factor (M-CSF), transforming growth factor-β (TGF-β), calcitriol, RANKL and macrophage inflammatory protein-1α (MIP-1α) [12]. 

1,25-dihydroxyvitamin D_3_ (1,25-(OH)_2_ vitamin D_3_, calcitriol): Vitamin D is a precursor that is first activated by hydroxylation in the liver to 25-(OH)_2_ vitamin D_3_ and then to 1,25-(OH)_2_ vitamin D_3_ (calcitriol) in the kidney by renal 1α-hydroxylase [60]. Calcitriol is the active form of vitamin D and increases plasma calcium by stimulating the absorption of dietary calcium in the intestines, increasing osteoclastic bone resorption when in high concentrations and augmenting the ability of parathyroid hormone to enhance renal calcium reabsorption in the kidney. Typically, in patients with HHM, calcitriol concentrations are low due to a compensatory response to the pathologic increases in serum ionized calcium. In contrast, increased serum calcitriol concentrations in patients with HHM have been recognized as an important humoral factor responsible for HHM in patients with non-Hodgkin’s and Hodgkin’s lymphoma [21,22]. Increased expression of the 1-α-hydroxylase enzyme that forms calcitriol has been identified in lymphoma cells, which has been speculated to contribute to the hypercalcemia in some patients with Hodgkin’s disease and malignant histiocytosis [61]. 

Macrophage inflammatory protein-1α (MIP-1α): MIP-1α is a cytokine that binds to chemokine G-protein coupled receptors, CCR1, CCR5 and CCR9 that are expressed on several cell types, including bone marrow stromal cells. MIP-1α activity has also been described as a chemotactic factor for osteoclasts and osteoclast precursors [62]. Increased concentrations of MIP-1α have been identified as clinical biomarkers in patients with adult T-cell leukemia/lymphoma (ATL), an aggressive hematological malignancy, and HHM occurs in >80% of patients [20]. MIP-1α stimulates calcium release from fetal mouse long bones [20]. One mechanism by which MIP-1α induces hypercalcemia in ATL patients is by upregulating RANKL on ATL cells [20]. It is unknown how MIP-1α stimulates osteoclastogenesis and HHM in other cancers. RANKL-dependent and independent mechanisms have been proposed [63,64]. 

Tumor necrosis factor-α (TNF-α): TNF-α is a pro-inflammatory cytokine that is critical to the pathogenesis of several neoplastic and inflammatory diseases characterized by increased osteoclastic bone resorption [65]. Constitutive overexpression and secretion of TNF-α by Chinese hamster ovarian cells resulted in increased osteoclastic bone resorption and HHM in mice, whereas bone resorption or hypercalcemia did not occur with control cells [66]. TNF-α stimulates osteoclastogenesis at two distinct time points in vivo. Early effects are due to priming of osteoclast progenitors in the bone marrow through RANKL-independent mechanisms. This increases the number of bone marrow osteoclast precursors by stimulating a proliferative response to M-CSF. The osteoclast precursors enter circulation and deposit at sites of bone resorption and complete differentiation into mature osteoclasts in the presence of RANKL [67]. The expression of RANKL is upregulated in surrounding osteoblasts, bone stromal cells and lymphocytes by TNF-α and binds to RANK on osteoclasts to promote increased osteoclast differentiation and bone resorption. In addition to stimulating systemic osteoclastic activity and bone resorption, the role of TNF-α as a sole agent that induces HHM remains obscure. Elevated levels of TNF-α have been identified in patients with HHM in the absence of PTHrP, suggesting it should be further investigated as a single mediator of HHM. However, synergistic and/or cooperative actions with PTHrP and other cytokines are primarily thought to result in TNF-α-associated HHM [15,68]. 

Interleukin-6 (IL-6): Increased concentrations of IL-6 have been identified in hypercalcemic patients with multiple myeloma, squamous cell carcinoma and ATLL. The mechanism by which IL-6 stimulates osteoclastic bone resorption is independent of RANKL. IL-6 recruits osteoclasts by activating signal transducer and activator of transcription (STAT)-1/3 and mitogen-activated protein kinase (MAPK) signaling pathways in osteoblasts [69]. Increased circulating IL-6 has been reported in hypercalcemic and normocalcemic patients and does not correlate with the severity of hypercalcemia. Therefore, IL-6 likely serves a synergistic or additive effect in the development of HHM [70]. This is supported by the observation that Chinese hamster ovarian cells overexpressing both IL-6 and PTHrP, in contrast to those with IL-6 overexpression alone, had marked hypercalcemia [71].

Interleukin-1 (IL-1): The IL-1 family consists of several cytokines involved in regulating inflammation and immunity. Circulating IL-1 has been observed in patients with hypercalcemia and pathologic bone loss due to increase osteoclast number and activity [16,72,73]. IL-1α and IL-1β are potent stimulators of osteoclastic bone resorption in vitro and in vivo [74,75,76,77]. Subcutaneous continuous infusions of IL-1α and -β into normal Swiss female mice resulted in a marked, dose-dependent increase in plasma calcium concentrations that was associated with osteoclastic bone resorption [77]. IL-1 is involved in the regulation of osteoclast differentiation, multinucleation and survival through intracellular signaling mediated by the IL-1 receptor (IL-1R1) expressed on maturing osteoclasts. Permissive levels of RANKL are necessary for IL-1 to induce osteoclast differentiation. IL-1 also acts on the cells of the bone microenvironment to stimulate osteoclast activity by inducing the expression of RANKL, macrophage-colony stimulating factor (M-CSF) and prostaglandin E_2_ by osteoblasts [78,79,80]. Increased reabsorption of calcium by the kidney has also been shown to contribute to hypercalcemia in mice and rats infused with continuous IL-1 [76,81]. 

Other cytokines: Several cytokines have been identified as factors involved in the pathogenesis of CAH. Cytokine profiles differ among patient with hypercalcemia associated with various malignancies. IL-1, IL-3, IL-11, M-CSF, GM-CSF, TNF-β, among others, have been proposed to serve as contributors to HHM. Serum from patients with HHM usually reveal increased concentrations of one or more of these factors, which result in a disproportionate rate of osteoclastic bone resorption to bone formation, breakdown of the mineralized matrix and release of calcium into systemic circulation.

### 1.2. Local Osteolytic Hypercalcemia

**Figure 2:**
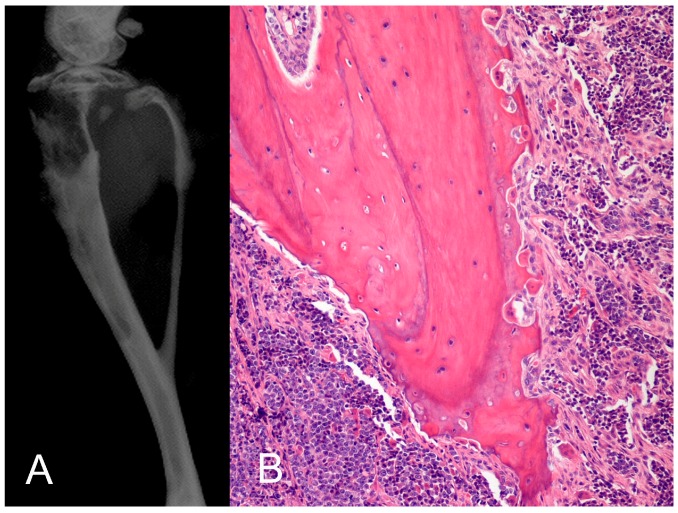
PTHrP-induced pathologic osteoclastic bone resorption. (**A**) The human adult T-cell leukemia cell line (RVATL) that secretes PTHrP was injected into the tibia of immunosuppressed mice and induced marked pathologic bone resorption observed radiographically; (**B**) histopathology revealed increased numbers of hypertrophic, active osteoclasts and extensive eroded bone surfaces. When mice were injected intraperitoneally with RVATL cells, there was increased circulating PTHrP and hypercalcemia due to HHM. This animal models support a dual endocrine and paracrine role of PTHrP in pathologic bone resorption and hypercalcemia.

Local osteolytic bone resorption is caused by primary bone malignancies, such as multiple myeloma, or bone metastases that secrete paracrine factors, which act to stimulate osteoclast function and local bone resorption. The degree of hypercalcemia in these cases correlates with the degree of tumor burden and can be severe in cases of advanced disease [18]. The cytokines and osteoclastogenic factors listed above and in Table 2 also are secreted by tumor cells and have local effects on bone cells and stimulate osteoclastogenesis. It is important to recognize that the tumor factors that induce LOH can also contribute to hypercalcemia in a humoral manner. Therefore, LOH and HHM can coexist in the same patient. PTHrP was originally thought to only play a role in HHM; however, in normocalcemic patients, PTHrP was also found in local bone neoplasms and metastasis that caused local osteolysis in normocalcemic patients [82]. This confirmed the important paracrine role of PTHrP, since it induces local osteoclastic bone resorption when primary cancers or metastases are present in bone (Figure 2) [83,84]. This is particularly common in women with breast cancer, since 80% of breast cancer bone metastases express increased PTHrP in the bone microenvironment. At sites of bone metastasis, locally-acting PTHrP upregulates RANKL-RANK signaling, which results in pathologic bone resorption and the release of calcium and other growth factors, such as TGF-β, from the mineralized matrix of bone that upregulate PTHrP gene expression and promote cancer progression [85]. This has been called the “vicious cycle” of bone metastasis. In metastatic breast cancer, hypercalcemia is infrequent, despite a high incidence of local PTHrP expression and bone resorption. Patients with bone metastases of carcinomas are usually normocalcemic, because the degree of pathologic bone lysis is insufficient to overcome the efficient homeostatic regulation of serum calcium. Plasma PTHrP concentrations are greater in patients with breast cancer bone metastases and hypercalcemia when compared to early breast cancer patients and those with bone metastases and normal serum calcium concentrations, suggesting both a paracrine and endocrine role for PTHrP in breast cancer-associated hypercalcemia [14]. 

In multiple myeloma, hypercalcemia is the most frequent metabolic complication and results from excessive local osteolysis and extensive tumor-induced bone resorption [17]. Myeloma cell secretion of RANKL, PTHrP, MIP-1α and TNFs (discussed above for HHM) act locally to enhance pathologic osteolysis. MIP-1α enhances osteoclast formation induced by IL-6, PTHrP and RANKL [63]. In addition to stimulating osteoclastogenesis, MIP-1α facilitates the survival of myeloma cells and increases their adhesion to bone [4,63]. 

### 1.3. Animal Models of HHM and LOH

HHM and LOH cause substantial patient morbidity and negatively impact patient prognosis and response to therapy. Similarly, veterinarians are faced with the challenge of implementing appropriate therapy and management of CAH in dogs and other animals. Because patients suffering from CAH are usually severely ill, efforts to investigate the underlying pathophysiologic mechanisms in affected patients are limited. Therefore, there has been much interest in using animal models and in vitro systems to identify the role of tumor-secreted factors involved in the pathogenesis of CAH. Identifying and characterizing the molecular and pathologic mechanisms of CAH are necessary for the development of novel adjuvant therapies and improved patient care. Spontaneous and experimental animal models have been invaluable to investigating these mechanisms. Examples of animal models include: (1) spontaneous cancers in domestic animals; (2) allogenic transplantation of rodent cancers; (3) xenogeneic transplantation of human and animal cancers to immunodeficient rodents; (4) drug-induced cancer in rodents; and (5) in vivo infusion of cancer-related humoral/paracrine factors responsible for CAH [84]. No single model can recapitulate all aspects of HHM or LOH; however, the information from the animal models has advanced the treatment of CAH and improved the understanding of the pathogenic mechanisms. Researchers should select the most appropriate animal model to investigate the specific question of interest. Additionally, while most models share in vivo features of the human syndrome, there are notable differences that must be considered. For example, rodent models with human or canine cancer xenografts and CAH consistently have high levels of serum calcitriol that contribute to CAH to varying degrees; however, humans with CAH usually have low serum calcitriol concentrations [45,86,87]. This review focuses on the comparative aspects of HHM and LOH in humans and animals, with a discussion of the available animal models for CAH research. 

### 1.4. Spontaneous and Experimentally-Induced Animal Models of HHM and LOH

#### 1.4.1. Spontaneous CAH in Dogs and Experimental Models

Cancer is the most common cause of hypercalcemia in dogs and accounts for 45%–65% of cases [30]. Lymphoma and adenocarcinoma of the apocrine gland of the anal sac are the most common causes of CAH; however, it also occurs with thyroid carcinoma, thymoma, multiple myeloma, various bone tumors, squamous cell carcinoma, mammary gland carcinoma, melanoma, primary lung cancers, chronic lymphocytic leukemia, renal angiomyxoma, nasal carcinoma, renal cell carcinoma, among other less common malignancies [28,29,31,32,88]. Dogs with hypercalcemia have increased total serum calcium, often greater than 12 mg/dL and an increase in ionized calcium (>1.3 mmol/L) [30]. The clinical signs of hypercalcemia can be nonspecific and are independent of the etiology. Clinical signs of hypercalcemia can be insidious if the increase is minimal or slow. Dogs with more severe elevations in serum calcium have lethargy, weakness, anorexia, vomiting, diarrhea, constipation, polyuria, polydipsia and can even progress to fatal renal failure [30]. 

The dog as a spontaneous model of human cancer has provided a valuable perspective that is distinct from studying human tissues or rodent models alone. The use of dogs as large animal, naturally-occurring models of disease has advanced our understanding of cancer pathogenesis, progression and therapy [89,90]. Several examples of CAH in dogs exist. These natural models have been used to investigate the pathogenesis of cancer and the development of novel therapeutics to advance both animal and human health [89]. Similarities exist between human and canine CAH, which demonstrates that the dog serves as an important large animal model for this syndrome [91]. Clinical trials in canine patients can advance treatment strategies for CAH and expedite development of novel therapies for both dogs and humans. Dogs with CAH may have advanced disease, which will preclude early studies to identify preventative strategies to mitigate hypercalcemia and its deleterious effects. Controlled clinical studies designed to investigate factors involved in the pathogenesis of CAH in dogs and novel treatment strategies can be expensive and challenging to recruit patients. Financial support and incentives for the owner are typical. However, the translational potential of using the dog as a large animal model of CAH is very promising. 

Adenocarcinoma of the apocrine glands of the anal sac: Adenocarcinoma of the apocrine gland of the anal sac (AGAC) is a tumor of older dogs that occurs at a similar frequency in females and males, which has a high rate of metastasis, but infrequently to bone [26]. Up to 50–70% of dogs with AGAC develop HHM characterized by increased serum calcium, decreased serum phosphorous and increased circulating PTHrP that correlates with serum calcium levels [25,92]. There is no apparent relationship between HHM and median survival time [93]. The development and characterization of a mouse model of canine AGAC (CAC-8) has been described [25,27,92,94]. This tumor (CAC-8) originated from a dog with AGAC and hypercalcemia and results in a subcutaneous tumor when injected into nude mice. Mice with CAC-8 develop hypercalcemia, hypophosphatemia, increased serum calcitriol and increased circulating PTHrP. Urinary excretion of calcium, cAMP and hydroxyproline and the rate of bone resorption and bone formation were increased. Histomorphometric analysis of the lumbar vertebrae revealed increased osteoid, resorptive surface and number of osteoclasts [25,27,84]. Intestinal calcium absorption or decreased urinary calcium excretion could not completely explain the HHM in these mice. In this animal model, PTHrP was the principal hypercalcemic factor; however, the cosecretion of transforming growth factors may also play a role in the development of HHM [94]. 

Lymphoma: Canine lymphoma is a naturally-occurring cancer that closely resembles the biologic behavior of the human disease. Approximately 40% of dogs with T-cell lymphoma develop HHM or a combination of LOH and HHM due to lymphoma in bone [19,23,24]. Biochemistry changes include increased serum calcium concentrations and normal to low serum phosphorous [95]. Serum calcitriol concentrations are decreased, normal or increased [25]. In humans with Hodgkin’s lymphoma and non-Hodgkin’s lymphoma (NHL) with HHM, serum calcitriol levels are frequently increased and have been implicated to play a significant role in this syndrome [21,22,96]. Increased calcitriol concentrations may also play a role in CAH in dogs with lymphoma, because a subset of canine patients had increased serum calcitriol concentrations [25]. However, this has not been a consistent finding in dogs with lymphoma and HHM [19,97]. Several studies have examined the association between PTHrP and lymphoma-associated CAH in dogs [24,25,98]. Increased plasma PTHrP concentrations occur in dogs with lymphoma and HHM, but plasma PTHrP does not always correlate with the degree of hypercalcemia [25,99]. Increased plasma PTHrP measured by an immunoradiometric assay (6.1 pmol/L) was described in one dog with lymphoma and hypercalcemia and no detectable plasma PTHrP (<1.1 pmol/L) in dogs with lymphoma and normocalcemia and healthy dogs. In addition, PTHrP mRNA expression was reported in one dog with lymphoma and hypercalcemia and four dogs with lymphoma and normocalcemia [98]. These findings indicate that additional tumor-related cytokines, in addition to PTHrP, likely play a role in lymphoma-associated CAH [25]. In dogs with T-cell lymphoma and hypercalcemia, PTHrP mRNA was upregulated to varying degrees in all of the hypercalcemic dogs with lymphoma [24]. TNF-α, a proinflammatory cytokine that stimulates osteoclastogenesis through RANKL-dependent and independent pathways, was upregulated or downregulated. RANKL, another osteoclast stimulatory factor that is secreted by activated T lymphocytes, osteoblasts and bone marrow stromal cells, was increased in 3/6 hypercalcemic lymphoma [24]. Interestingly, IL-6, a proinflammatory cytokine implicated in the pathogenesis of HHM, was downregulated in all samples [100]. Unlike the primary role of PTHrP in HHM due to adenocarcinoma of the apocrine glands of the anal sac, PTHrP likely plays a cooperative or synergistic role with other cytokines in dogs with lymphoma and HHM. 

A xenograft mouse model of canine T-cell lymphoma with HHM was developed to investigate the humoral factors and cytokines involved with the disease [24]. The cells were derived from a canine patient with lymphoma and hypercalcemia and resulted in multicentric lymphoma when xenografted intraperitoneally into NOD/SCID mice. The proposed mechanism of hypercalcemia was upregulation of tumor-produced PTHrP and TNF-α. Bone histomorphometry demonstrated that there was increased bone resorption, similar to what occurs in both humans and dogs with HHM. Future work using this model will provide valuable insight into the role of individual and synergistic cytokines in the development of HHM in dogs with T-cell lymphoma. 

Multiple myeloma: Multiple myeloma (MM) is an uncommon plasma cell malignancy of older dogs that primarily involves bone and is associated with hypercalcemia in 15%–20% of cases [101]. Hypercalcemia is regarded as a poor prognostic factor in canine MM and is the result of several mechanisms similar to humans with MM-associated CAH [102]. These include extensive local tumor-induced bone resorption, secretion of PTHrP and/or an increase in M-protein-bound calcium. In humans with MM, more than 88% of patients develop extensive osteolytic bone disease and hypercalcemia [103]. Radiographic evidence of osteolysis was identified in 50% of canine patients; however, not all cases with bone resorption result in hypercalcemia [101,104]. Additional tumor-mediated mechanisms are responsible for stimulating osteoclasts in human MM, including IL-1, IL-6, tumor necrosis factor-α (TNF-α), TNF-β (lymphotoxin), RANKL, MIP-1α and PTHrP, among others [103]. In canine MM, increased PTHrP expression has been speculated to serve a role in CAH; however, some report undetectable circulating PTHrP [101,105,106]. Serum calcium exists in three fractions; ionized, complexed and protein bound. In human MM, negatively-charged myeloma proteins will bind to calcium resulting in an increase in total calcium concentrations, but no increase in serum ionized calcium [107]. This mechanism has been reported in a dog with MM and a monoclonal gammopathy [106]. 

#### 1.4.2. Spontaneous CAH in Cats and Experimental Models

Cancer as a cause of hypercalcemia in cats accounts for up to 30% of cats with hypercalcemia [33,34]. In cats, hypercalcemia occurs with lymphoproliferative disorders, squamous cell carcinoma (SCC), multiple myeloma, osteosarcoma, fibrosarcoma, bronchogenic carcinoma and other malignancies [35,37,40,108]. Lymphoma and SCC are the most common causes. The clinical signs of hypercalcemia in cats are similar to those observed in dogs. The more common cancers associated with CAH in cats are described below. The advantage of using cats is they represent a large animal model of the disease. Spontaneous cancers in cats associated with hypercalcemia and the mechanisms by which they occur are similar to those in humans. However, CAH in cats is rare, and special attention to species-specific drug metabolism must be considered in therapeutic trials with cats, which can limit their translatability to humans.

Squamous cell carcinoma: Similarities exist between feline and human oral squamous cell carcinoma (OSCC), including local aggressive osteolytic bone resorption and PTHrP expression. In humans, squamous cell carcinoma of the head, neck and oropharyngeal region is a well-recognized cause of hypercalcemia, and tumors are often characterized by high PTHrP expression [108]. The majority of human cases with SCC and CAH are humorally mediated [108]. A similar mechanism may exist in cats. In a previous report of cats with SCC and CAH, osteolytic lesions were not detected [34,35]. Furthermore, increased circulating concentrations of PTHrP have been detected in cats with SCC-associated CAH [39]. Twenty five percent of humans with invasive head-and-neck SCC have hypercalcemia secondary to tumor-derived factors that increase local osteoclast-mediated bone resorption, rather than humoral mechanisms acting on bone distant from the primary tumor [109]. Calcium released from local bone resorption due to tumor invasion has been proposed as a mechanism to increase serum calcium in some cases of human SCC-associated CAH [110,111]. A similar mechanism may exist in feline oral SCC (OSCC) with CAH [112,113,114,115]. Both human and feline OSCC cell lines express high levels of osteoclast-stimulating factors, such as PTHrP, IL-6, IL-8 and IL-11 [114,115]. Martin et al. characterized the bone resorptive activity in a preclinical mouse model of bone-invasive feline OSCC [114]. The cell line derived from a feline SCC (SCCF2) resulted in maxillary bone invasion and osteoclastic bone resorption. Furthermore, the cells synthesized high levels of PTHrP mRNA, as was secreted PTHrP and stimulated osteoclastic bone resorption. Messenger RNA expression of RANKL, a potent inducer of osteoclast activity, was low in the SCCF2 cells. However, RANKL mRNA expression in murine pre-osteoblasts was upregulated when co-cultured with SCCF2 cells. These results suggest that SCCF2 expression of PTHrP plays an indirect role in stimulating pathologic osteolysis in OSCC-associated pathologic bone resorption. Therefore, PTHrP likely plays an important role in local tumor-mediated osteolysis and HHM in OSCC. 

Lymphoma: Hypercalcemia has been associated with lymphoproliferative neoplasia in cats [34,36,37,38]. In a previous report, 82% of cats with neoplasia and hypercalcemia were due to PTH-independent mechanisms, and circulating PTHrP was increased in the majority of cases [39]. Increased serum PTHrP has been reported in cats with lymphoma and CAH. Cats with feline leukemia virus (FeLV)-associated leukemia-lymphoma can have hypercalcemia in the absence of skeletal metastasis and no renal or parathyroid pathology. The findings in this study suggest that humorally-mediated mechanisms were responsible for the development of hypercalcemia [39]. The mechanism of hypercalcemia in FeLV-induced lymphoma/leukemia in cats is unknown, but it may be similar to HHM in humans with human T-cell lymphotropic virus type 1 (HTLV-1)-induced lymphoma/leukemia, which is due to increased serum PTHrP and possibly synergistic cytokines. 

#### 1.4.3. Spontaneous CAH in Horses

Spontaneous CAH occurs in horses, but at a lower frequency than in humans and dogs [45]. HHM is a well-recognized equine paraneoplastic syndrome, which occurs in 25% of horses with gastric SCC [42]. The types of CAH-associated neoplasms are similar in horses to those of other species. HHM is likely the pathogenesis of hypercalcemia in most horses with CAH, since there are usually no osteolytic lesions or bone metastases, and there is a lack of parathyroid and renal pathology. Increased serum PTHrP has been reported and is likely an important mediator of equine HHM. Increased serum PTHrP concentrations have been documented in horses with CAH associated with MM and ameloblastoma [42,43,44,45,116]. Additional humoral mediators of CAH have been identified, including tumor-derived prostaglandins [41]. 

#### 1.4.4. Spontaneous CAH in Exotic Species

Hypercalcemia of malignancy has been reported in Amazon parrots with malignant lymphoma [117]. 

#### 1.4.5. Spontaneous and Experimentally-Induced CAH in Rodent Models

Rodent models have greatly advanced our understanding of CAH. Several strains of mice, rats and rabbits have been used to isolate and identify humoral and paracrine factors involved in the pathogenesis of CAH and investigate the pathophysiology of hypercalcemia in vivo. In addition, animal models have been instrumental in preclinical drug trials and the testing of novel therapeutics useful for patients with CAH. Investigation of CAH in rodent models involves four major methods: (1) allogenic transplantation of tumors; (2) xenogenic transplantation of tumors; (3) drug-induced tumor development; and (4) infusion of potential factor/factors responsible for CAH [84]. The use of each method varies between rodent species and the research hypothesis. It is also important to consider the advantages and disadvantages of selecting each animal model. Using rodent models has several general advantages, including they are less expensive, often readily available, have similar pathophysiologic mechanisms compared to humans and there are multiple strains to choose from that have been well characterized. Syngeneic transplantation of cancer cells or solid tumors resulting in CAH are advantageous because the host immune system will be competent, which more closely recapitulates the natural tumor, microenvironment and host response. The disadvantage is that there are few syngeneic rodent models of CAH, and they may not accurately represent the human condition. Xenogenic transplantation of tumors is relatively easy to perform, and multiple cell lines have been described from several species. Human cancer cells or tissue implanted into immunosuppressed mice often recapitulate the original human cancers, but the metastatic pattern may be absent or different. Disadvantages include the lack of host immunity against the cancer, which is an important component in spontaneous human cancer. This is particularly important in immunosuppressed rodents that have a knockout of one or more cytokines, which may play an integral role in the pathogenesis of CAH. It is interesting to note that athymic (nude) mice may have a robust B lymphocyte and innate macrophage response to cancer xenografts; whereas their T lymphocyte response is minimal. Modeling carcinogenesis using direct exposure to carcinogens enables investigations of premalignant lesions and the early pathogenesis of CAH. Chemical-induced tumor development also allows the use of an immunocompetent host. The disadvantages include the prolonged time for tumor development, laborious application procedures and potentially hazardous chemicals. Direct infusion of single or multiple CAH-inducing factors is an excellent method to model CAH and investigate the interactions of factors in the pathogenesis of the disease. Disadvantages include the challenges of administration using catheters or implantable infusion pumps and the limited duration of experiments. 

## 2. Mouse Models of CAH

The majority of mouse models of CAH are derived from xenograft transplantation (Table 3). Either primary cancers or cancer cell lines have been used. The host species for the cancers include humans, dogs, cats, hamsters and other species. Few allogenic models have been developed and include the HSDM1 fibrosarcoma, the CE murine mammary carcinoma and the 5T multiple myeloma (MM) model. Additional techniques used to study the pathogenesis of HHM include the infusion of bioactive molecules (e.g., synthetic PTHrP) and the induction of squamous cell carcinoma by cutaneous application of dimethylbenzanthracene [50,118]. 

Allogenic models: The allogenic HSDM1 fibrosarcoma was derived from a mouse and results in progressive hypercalcemia 2–3 weeks following serial transfer into Swiss albino mice [119]. Evidence suggests that prostaglandin E_2_ (PGE_2_) is responsible for mediating the hypercalcemia, which is a unique, but uncommon mechanism of HHM. Increased serum calcium concentrations were prevented by treatment with hydrocortisone and indomethacin, but not a low calcium diet [149,150]. Additional humoral factors and cytokines have not been described in this model, and it is unknown whether PGE_2_ is solely capable of inducing the hypercalcemia.

The murine mammary carcinoma (CE maca) is a spontaneously derived cancer from the BALB/c mouse that can be transplanted to recipient mice and results in marked granulocytosis and hypercalcemia 3–4 weeks following subcutaneous or intramuscular injection [120,121]. Serum calcium concentrations return to the reference range following tumor removal. In this model, hypercalcemia is associated with a significant increase in urinary hydroxyproline, hypercalciuria and an increase in urinary cAMP excretion [151,152]. Increased intestinal calcium absorption as the cause of hypercalcemia was excluded based on the finding that the hypercalcemia was not corrected with a calcium-deficient diet [152]. Marrow granulocytic hyperplasia is associated with an increase in the number of osteoclasts lining endosteal surfaces and increased bone resorption [151,152]. Granulocyte colony stimulating factor (G-CSF) and macrophage colony stimulating factor (M-CSF) were detected by Northern blot analysis in CE maca tumors and treatment with anti-M-CSF reduced the neutrophilia, but did not affect the hypercalcemia [153]. Granulocytic hyperplasia has been associated with increased osteoclasts and osteoclast activity [154]. It could be speculated that the increased granulocytic hematopoiesis involves cytokines that could indirectly influence neighboring bone cells to promote osteoclastogenesis. 

The 5TMM cell lines were derived from aged inbred C57BL/KalwRij mice with spontaneous multiple myeloma (MM). These tumor lines have been extensively characterized and are useful to investigate the pathogenesis of MM, including CAH and associated osteolytic lesions. When sub-clones of the 5T2MM and 5T33MM cell lines are injected intravenously into mice, hypercalcemia and osteolytic bone lesions are observed [17,122,123]. The tumor cells express IL-6, which is an important factor in the development of HHM. Inhibition of CCR1 and CCR5 receptors and their master ligand, MIP-1α, reduce osteolysis and bone resorption in vivo in the 5TMM model [155]. 

Transgenic model: A subset of HTLV-1 infected humans will develop adult T-cell leukemia/lymphoma, and HHM occurs in 50%–90% of patients. HTLV1 encodes for two viral oncogenes, Tax and HBZ, with Tax being more extensively studied. Tax is a transcriptional activator that can transactivate several cellular and viral genes and includes genes involved in CAH (e.g., IL-1, IL-6, TNF-α, and PTHrP). An HTLV-1 Tax+ transgenic mouse model has been developed to study the roles of these factors in vivo. Under the human granzyme-B promoter (expressed in cytotoxic T-cells and natural killer cells), Tax+ transgenic mice develop spontaneous ear and tail tumors associated with hypercalcemia and vertebral osteolytic bone lesions [124]. Tax+ tumors express several factors, including macrophage colony stimulating factor (M-CSF), IL-1, IL-6, TGF-β, TNF-α, MIP1α, RANKL and PTHrP [124]. These factors are known to have a role in the development of CAH through both humoral mechanisms and local stimulation of osteoclastogenesis and bone resorption. 

Xenograft mouse models: Many human and animal cancer cell lines have been used in immunosuppressed mice to recapitulate and investigate the pathogenesis of HHM in vivo. Cancer cells are most often implanted in the subcutis, but other routes of administration include intraperitoneal, intratibial, intracardiac, orthotopic and intravenous. 

Lung cancers: HARA, RWGT2, BEN, Lu-65A, Lu-61, OKa-C-1: In humans, hypercalcemia occurs in 8%–12% of patients with advanced lung cancer, with an incidence of up to 23% in patients with the pulmonary squamous cell carcinoma [13]. To recapitulate HHM in vivo, several human-derived cancer cell lines have been transplanted into immunodeficient mice. Mice bearing RWGT2, Lu-61, HARA and/or BEN xenografts develop HHM, and the tumor cells have high PTHrP expression. Hypercalcemia induced by RWGT2 is lowered in tumor-bearing mice following administration of IL-6 neutralizing antibodies. Inhibition of RANK decreases serum calcium in mice with RWGT2 tumors despite no change in circulating PTHrP concentrations [127,128,129,156]. The HARA cell line was derived from a human squamous cell lung carcinoma that did not have evidence of bone metastases, but had increased serum PTHrP and calcium concentrations. The HARA cells express high levels of PTHrP mRNA and following left ventricular intracardiac injection result in bone metastasis and high levels of serum PTHrP and calcium, similar to the syndrome observed in human patients. In this model, PTHrP plays a role in both HHM and local bone resorption at sites of bone metastasis [157]. The HARA-B cell line was established from a bone metastasis that developed following intracardiac injection of HARA cells. Nude mice bearing HARA-B tumors develop cachexia, hypercalcemia and increased serum PTHrP 3–4 weeks following inoculation. Combined therapy with OPG and anti-PTHrP antibodies reversed the hypercalcemia in these mice [158]. The role of the calcium sensing receptor (CaR) in lung SCC was explored using a xenograft model of BEN SCC. Rapid development of HHM in BEN mice was the result of PTHrP secretion by the cancer cells. In addition, extracellular calcium signaling through CaR was found to increase PTHrP production by stimulating the MAPK pathway. Therefore, hypercalcemia may be responsible for a positive feedback loop to induce increased PTHrP production by the cancer cells [159]. The Lu-65 cell line was derived from a patient with an anaplastic lung cancer and caused increased bone resorption and hypercalcemia in vivo. The Lu-65 cells produce and secrete large amounts of prostaglandin E_2_, which may be responsible for the increased bone resorption and HHM [160]. 

Squamous cell cancers: T3M-1, MH-85, OKa-N-1: The incidence of HHM in squamous cell carcinoma (SCC) of the head and neck, oral cavity and skin has been reported in up to 50% of cancers, with the majority being humorally mediated [161]. Several cell lines have been derived from these cancers and implanted into nude mice to investigate the mechanisms of circulating tumor factors that result in hypercalcemia. Leukocytosis, cachexia and hypercalcemia have been observed in patients with SCC and are reproduced in several animal models engrafted with cell lines from patients. The T3M-1 cell line was established from a human SCC-bearing nude mouse that developed marked leukocytosis and hypercalcemia. The cells express mRNA for both PTHrP and granulocyte colony stimulating factor (G-CSF), which mediate the paraneoplastic changes. Additionally, IL-1α may be, in part, responsible for the HHM [130]. MH-85 cells cause hypercalcemia, leukocytosis and cachexia in tumor-bearing nude mice [128]. The OKa-N-1 cell line is a squamous cell carcinoma cell line derived from a patient with leukocytosis and hypercalcemia. Following subcutaneous injection, nude mice develop cachexia, leukocytosis and hypercalcemia, and tumors express G-CSF and PTHrP mRNA [162]. The relationship between G-CSF and PTHrP in these cancers is uncertain. 

Renal carcinoma: 786-0: Nude mice with subcutaneous xenografts of the human renal carcinoma cell line, 786-0, develop hypercalcemia, hypophosphatemia and increased serum calcitriol. Urinary cAMP excretion was unchanged, which is usually increased in patients with HHM [132]. Following resection of the subcutaneous tumor, serum calcium concentrations returned to the normal range, consistent with a humoral mechanism for the hypercalcemia. A circulating tumor factor (likely PTHrP) resulted in the increased serum calcium and was associated with increased bone resorption and high bone turnover [131].

Pancreatic cancer: FA-6: The FA-6 cell line was derived from a human pancreatic cancer and resulted in HHM due to the production of PTH-like (likely PTHrP) and TGF-α activities after being injected subcutaneously in nude mice. These factors caused increased osteoclastic bone resorption [139].

Ovarian cancer: Small cell ovarian carcinoma of the hypercalcemic type (SCCOHT-1): The SCCOHT-1 cell line was derived from a tumor of a 31-year-old woman with a small cell ovarian carcinoma of the hypercalcemic type [142]. After subcutaneous injection, female NOD/scid mice developed subcutaneous tumors and hypercalcemia. Interestingly, when the combination of calcium and epothilone B, an inhibitor of mitosis was administered subcutaneously to the hypercalcemic mice, serum calcium concentration returned to normal; however, there was no effect with either agent alone [148]. Increased calcium levels can result in cell death in ovarian cancer cells in vitro, and hypercalcemia may represent a mechanism to antagonize tumor growth. The effects of hypercalcemia may synergize with epothilone B, however, remain unknown. 

Urothelial cancer: Transitional Cell Carcinoma (TCC): Patients with urothelial cancers may develop HHM. The human TCC cell line causes HHM in nude mice when injected subcutaneously [133]. When conditioned medium was collected and added to neonatal mouse calvaria, marked bone resorption occurred in vitro. The mechanism of bone resorption by the TCC cells was a macromolecular secreted factor that stimulated prostaglandin synthesis by bone cells.

Esophageal carcinoma: KEsC-2, EC-GI: Subcutaneous injections of nude mice with human KEsC-2 cells derived from a patient with esophageal carcinoma, caused HHM, and the cancer cells expressed PTHrP mRNA [134]. In addition, EC-GI ( a cell line derived from a human patient with esophageal carcinoma and hypercalcemia) cancer xenograftsin nude mice resulted in HHM that was mediated by the synergistic actions of PTHrP and IL-1α [135].

Melanoma: SEKI, A375: The human-derived SEKI cell line caused HHM following subcutaneous injection in nude mice. The SEKI cells produced PTHrP mRNA and secreted biologically-active PTHrP protein [134]. The human A375 cell line caused osteolytic bone metastases and hypercalcemia following intracardiac injection in nude mice. Bone resorption at the sites of metastasis was attributed to increased expression of TGF-α [137]. Hypercalcemia also developed when the A375 cells were injected into the mammary fat pad of SCID mice despite a lack of bone metastases. The degree of hypercalcemia correlated with serum concentrations of PTHrP [136].

Mammary tumor: S180-A: The ascites sarcoma 180 is a transplantable human malignancy that results in HHM following subcutaneous injection in nude mice. The mechanism of HHM and increased bone resorption was independent of PTHrP and PGE_2_ and was mediated by TGF-α, IL-1α and IL-6 [138].

Colorectal cancer: C-26: The human C-26 cell line was derived from a colon adenocarcinoma, has high PTHrP expression and caused increased circulating PTHrP, hypercalcemia and increased osteoclastic bone resorption in normal mice (CDF1 mice) [140,141]. Blood ionized calcium concentrations returned to normal following treatment with the RANKL inhibitor, osteoprotegerin (OPG), and were reduced with the bisphosphonate, zoledronic acid. Histomorphometry of the tibia revealed a greater reduction in osteoclast surface and serum TRAP5C in mice treated with OPG compared to zoledronic acid [141].

Multiple myeloma: ARH-77, primary multiple myeloma patient-derived cells: Multiple myeloma is one of the most common causes of CAH in humans and is the result of extensive bone lysis, secretion of PTHrP and increased protein-bound serum calcium. ARH-77 cells are an IgG_k_-secreting human plasma cell leukemia cell line that mimics human myeloma when injected into SCID mice [146]. ARH-77 tumor-bearing mice develop hypercalcemia following intravenous injection that resulted from increased osteoclastic bone resorption. ARH-77 hypercalcemic mice did not have detectable TGF-α, IL-6, TNF-α or PTHrP mRNA expression or circulating plasma proteins. Furthermore, in vitro bone resorption was not inhibited following treatment with anti-IL-6 or TNF-β antibodies [3]. Dikkof-1 (DKK-1) was identified as a significant contributor to local bone loss in myeloma through the inhibition of osteoblasts. Following engraftment of primary multiple myeloma cells from patients with clinical disease into SCID-rab, anti-DKK1 therapy significantly increased bone mineral density, increased osteoblast number and reduced osteoclast TRAP staining [147]. 

Adult T-cell leukemia/lymphoma (ATL): RVATL, MET-1, primary ATL cells: Immunodeficient SCID mice injected intraperitoneally with primary human ATL cells develop hypercalcemia and increased serum PTHrP concentrations [145]. The RV-ATL cells were derived from an ATL patient [163] and, when injected intraperitoneally into SCID/beige or NOD/scid mice, developed multicentric lymphoma, ascites and HHM. The mice also had increased plasma PTHrP levels and increased osteoclastic bone resorption [143]. This model is an excellent tool to study the pathogenesis of ATL and HHM, as it closely recapitulates the disease observed in ATL patients. The RV-ATL cells were stably transduced with the luciferase gene for in vivo bioluminescent imaging. Tumor-bearing mice treated with zoledronic acid (a bisphosphonate) and PS-341 (a selective proteasome inhibitor) had decreased tumor burden and HHM [144]. NOD/scid mice injected intraperitoneally with the MET-1 ATL cells develop leukemia, multicentric lymphoma involving multiple organs and HHM. Tumor-bearing mice had increased plasma PTHrP concentrations, and the MET-1 cells expressed PTHrP and RANKL mRNA [164,165].

Several additional ATL primary tumors have been inoculated into nude mice to understand the mechanisms that regulate HHM in ATL [134,166]. These experiments have been instrumental to advance the discovery of new therapeutic agents, including vitamin D analogues, bisphosphonates, OPG, soluble RANKL and proteasome inhibitors, among others [127,140,144,158,167].

Chemical-induced model: Carcinogenic compounds have been directly applied to mice to investigate the induction of cancer development following exposure. When dimethylbenzanthracene was applied to the skin of female CD1 mice, ninety percent of the mice develop SCC and HHM. The mice had hypercalcemia, hypophosphatemia, hypercalciuria, increased serum calcitriol, increased urinary cAMP and increased bone resorption [118]. Tumor extracts contained a biologically-active protein, slightly larger than PTH, yet had similar activity to PTH. Given these findings, the HHM was likely mediated by PTHrP. 

Infusion of bioactive peptides and molecules: Infusion of synthetic peptides of PTHrP-(1–40, 1–34, 1–36) in both rat and mouse models has improved the understanding of the role PTHrP has in the pathogenesis of HHM [50,125,126,168]. Infusion of PTHrP in nude mice resulted in increased serum calcium and calcitriol, decreased serum phosphorous, increased urinary excretion of calcium, phosphorous and cAMP. Unlike the increased bone resorption observed in patients with HHM, there were increases in both bone resorption and bone formation in the mice [50]. Therefore, mouse models of HHM may have subtle differences compared to human patients. 

### 2.1. Rat Models of CAH

Allogenic models: Two spontaneously-derived, transplantable rat tumors, Rice H-500 and the Walker rat carcinosarcoma 256, have been developed as models of CAH [169,170,171,172,173]. The testicular tumor of Fischer rats (Rice H-500) does not metastasize following subcutaneous injection, and rats develop progressive hypercalcemia within 8–10 days with increased circulating PTHrP concentrations [174]. Additional clinicopathologic changes include hypophosphatemia, hypercalciuria, hyperphosphaturia and increased urine cyclic adenosine monophosphate (cAMP) excretion [169,173]. Osteoclastic bone resorption was increased, and bone formation was decreased. There was increased renal tubular reabsorption of calcium despite hypercalcemia and hypercalciuria, as seen in human patients with HHM. Renal tubular calcium reabsorption is thought to be necessary for the pathogenesis of HHM in this model. This is supported by the observation that serum calcium levels were increased despite inhibition of osteoclastic bone resorption following treatment with dichlorodimethylene bisphosphonate [175]. Furthermore, renal phosphate uptake was specifically and persistently inhibited, which contributed to the hypophosphatemia [176]. While serum PGE has been shown to parallel the increase in serum calcium in the Rice model, inhibition of PGE did not affect the degree of hypercalcemia [169]. 

Immunoreactive serum PTH levels were decreased, and PTHrP and calcitriol concentrations were increased in rats with H-500 tumors [169]. The Rice H-500 model was useful in the identification and purification of PTHrP and in the development of an immunoradiometric assay [177,178,179]. It has been demonstrated that PTHrP plays a central role in the development of HHM in this model. Plasma calcium levels were normalized for several days following passive intravenous immunization with antiserum to PTHrP. The use of this model has been useful to investigate the role of PTHrP and the calcium-sensing receptor (CaR) relationship. Treatment of H-500-bearing mice with cinacalcet (an allosteric activator of CaR) decreased hypercalcemia in a dose-dependent manner, but did not affect serum PTHrP. Plasma calcitonin levels decreased with the treatment-mediated decrease in ionized serum calcium [167]. The Rice H-500 model has been used to identify the role of additional factors that mediate HHM (e.g., transforming growth factors (TGFs)). Conditioned medium from tumor cell cultures and tumor extracts revealed EGFR binding with TGF immunoreactivity and an absence of EGF activity. A potential for TGF stimulation of bone resorption through EGFR binding was proposed in this model [179,180]. TGF-α binds to the epidermal growth factor (EGF) receptor, and both TGF-α and EGFs are capable of stimulating bone resorption through this receptor. 

The second allogenic rat model is the Walker 256 rat carcinosarcoma model. There are two variants of the Walker rat carcinosarcoma 256 and only one that results in hypercalcemia. The hypercalcemic variant is characterized by increased serum calcium, tartrate-resistant acid phosphatase, calcitriol and plasma PTHrP concentrations. Tumor-bearing rats had decreased calcium content in their bones secondary to PTHrP-mediated osteolysis [181]. Despite high serum calcium levels, the rats had a normal fractional rate of intestinal calcium absorption, suggesting an intestinal contribution to the hypercalcemia [182]. This model demonstrated a role of the TGF-β-PTHrP axis in HHM. Treatment of Walker carcinosarcoma 256 cells with TGF-β1 increased mRNA expression of PTHrP, but there was a decrease in secretion of PTHrP [183]. This suggests that the effect of TGF-β is mediated at the transcriptional level. The Walker carcinosarcoma model has been widely employed to test antihypercalcemic agents [184,185,186,187,188]. 

Several other rat models of HHM have been described (Table 4). Allogenic transplantation of a lung carcinoma line (IP) into female F344 rats resulted in HHM [189]. This model was used to elucidate the potential impact of age on HHM. Following intraperitoneal injection of the IP cell line into three different age groups of female Fisher rats, they developed hypercalcemia, increased osteoclastic bone resorption and increased circulating levels of PTHrP. The degree of HHM varied between the groups with the greatest difference being in tissue calcification. The incidence and severity was decreased to absent as mice aged. The mechanism of tissue calcium deposition as a result of HHM in this model may be influenced by age [189]. 

Xenogeneic models: Xenogeneic transplantation of human cancers into nude rats has been used to investigate HHM [190,191,192,193,194,195]. Human uterine carcinoma xenografts caused hypercalcemia, hypophosphatemia, increased urinary phosphate and cAMP secretion and suppressed serum calcitriol concentrations. HHM in this model has been speculated to be predominantly attributed to secreted humoral factors, such as PTHrP [191].

The PTHrP-producing human infantile fibrosarcoma (OMC-1) resulted in HHM following subcutaneous injection into nude rats. The effect of calcium and PTHrP in regulating calcitriol was examined in this model. OMC-1-bearing rats had severe hypercalcemia and decreased serum calcitriol concentrations. When rats are treated with bisphosphonate therapy, the calcium concentrations were restored with an accompanying increase in serum calcitriol levels. However, when the mice were treated with a neutralizing antibody to PTHrP, serum calcitriol levels remained low despite a reduction in serum calcium. 1α-hydroxylase is an enzyme in the kidney and is responsible for converting 25-h

ydroxyvitamin D into active vitamin D (calcitriol). 1α-hydroxylase levels in the kidney were reduced in OMC-1-bearing rats with severe hypercalcemia, but were increased following bisphosphonate therapy, and there was no change in 1α-hydroxylase expression following PTHrP treatment. The authors suggest that PTHrP regulation of 1α-hydroxylase was mediated by calcium concentrations, and hypercalcemia reduced serum calcitriol by suppressing PTHrP-induced 1α-hydroxylase expression [193]. Chemical-induced models: Intraperitoneal injection of nitrosourea compounds into Sprague-Dawley rats resulted in a 90% incidence of mammary gland carcinoma and hypercalcemia. The rats did not have bone involvement or evidence of bone metastasis, indicating a humoral mechanism, similar to what is observed in the pathogenesis of HHM in some women with breast cancer [192]. 

### 2.2. Rabbit Model of CAH

The VX-2 carcinoma developed from malignant transformation of a viral-induced Shope papilloma that caused HHM following transplantation into the hind limb of New Zealand white rabbits [196]. Following transplantation, the tumor grows rapidly with local infiltration and metastasis to regional lymph nodes and lungs. Humoral mechanisms are responsible for pathologic hypercalcemia. This was supported by an absence of bone metastasis, yet increased bone resorption was observed. Progressive hypercalcemia and hypophosphatemia occurred 2–4 weeks following tumor transplantation [197,198,199,200]. Serum PTH and urinary cAMP excretion did not change. Circulating PTHrP has not been reported. Histomorphometric analysis of trabecular bone at distant sites revealed a reduction in osteoblast activity and an increase in the osteoclast number and resorptive surfaces [197,201].

Conflicting reports have attempted to elucidate the mechanism for the HHM in this model. The VX-2 cells contain large amounts of prostaglandin E. Indomethacin (a nonsteroidal anti-inflammatory agent) decreased PGE_2_ and prevented the hypercalcemia. These results suggest that the HHM was due to tumor cell secretion of prostaglandin E_2_, resulting in generalized osteopenia and increased bone resorption [198,199]. However, dietary calcium restriction has been found to be more effective at normalizing plasma calcium levels then inhibiting PGE_2_. It was concluded that the hypercalcemia in this model was dependent on an increase in gastrointestinal calcium absorption rather than prostaglandin-mediated bone resorption [196].

## 3. Conclusions

Spontaneous and experimental animal models have significantly advanced cancer research and our knowledge of tumor biology and paraneoplastic syndromes. The use of animal models of CAH will improve our understanding of the pathogenesis of HHM and local tumor-induced bone resorption and facilitate novel drug discovery. Spontaneous HHM in dogs closely resembles the syndrome in humans and will serve as an excellent large animal model for future research and testing new therapeutics. While similarities exist between spontaneous and experimentally-induced models and the human syndrome, important differences exist. The investigator must be aware of the clinicopathologic differences observed between the models, as well as the strengths and limitations of each so that the appropriate model may be selected for experimental investigations. While several models are described in this report, future studies should focus on improving current models and the development of additional models with translatability to both humans and animals. Humanized mice, transgenic rodents and constitutive, conditional or temporal gene knock-in/knock-out of CAH factors in rodents could be used to elucidate the complexity of the pathogenesis of CAH. Clinical researchers should attempt to identify additional factors or cytokines that could potentially be involved in the pathogenesis of CAH. The use of clinical trials in dogs with spontaneous CAH is also needed to advance our understanding of the pathogenesis and treatment of this important disorder in animals and humans. 

## Figures and Tables

**Table 1:** Cancers associated with hypercalcemia (CAH) in humans and animals.

Species	Tumors Associated with CAH	Reference
Humans	Lung cancer	[2,5,7,13]
	Breast cancer	[2,5,7,14]
	Squamous cell carcinoma	[15,16]
	Multiple myeloma	[2,17,18]
	Adult T-cell lymphoma	[2,3,4,19,20]
	Less common: colorectal cancer, multiple endocrine neoplasia, prostate cancer, urinary tract cancers, pancreatic cancer, non-Hodgkin’s lymphoma	[2,7,21,22]
Dog	Lymphoma (usually T-cell type)	[19,23,24,25]
	Apocrine gland adenocarcinoma of the anal sac	[25,26,27]
	Multiple myeloma	[28,29,30]
	Less common: thyroid carcinoma, thymoma, various bone tumors, squamous cell carcinoma, mammary carcinoma/adenocarcinoma, melanoma, primary lung tumors, chronic lymphocytic leukemia, renal angiomyxoma, nasal carcinoma, renal cell carcinoma	[29,31,32]
Cat	Squamous cell carcinoma	[33,34,35]
	Lymphoma	[36,37,38]
	Multiple myeloma	[34,39]
	Less common: osteosarcoma, fibrosarcoma, bronchogenic carcinoma	[34,39,40]
Horse	Squamous cell carcinoma, especially gastric	[41,42]
	Lymphoma	[40]
	Less common: multiple myeloma, ameloblastoma	[43,44,45]

**Table 2:** Factors and cytokines involved in humoral hypercalcemia of malignancy (HHM) and local osteolytic hypercalcemia (LOH).

Factor	Mechanism
Parathyroid hormone-related protein (PTHrP)	HHM; LOH
Macrophage inflammatory protein-1α (MIP-1α)	HHM; LOH
Calcitriol (1,25-(OH)_2_ vitamin D_3_)	HHM
Receptor activator of nuclear factor-κB ligand (RANKL)	LOH
Interleukin-1 (IL-1), IL-3, IL-6, IL-8, IL-11	LOH; HHM
Tumor necrosis factor-α (TNF-α), TNF-β	LOH; HHM
Macrophage colony stimulating factor (M-CSF)	LOH; HHM
Granulocyte macrophage-colony stimulating factor (GM-CSF)	LOH; HHM
Various, less common cytokines (prostaglandin E2, TGF-α)	LOH; HHM

**Table 3:** Mouse models of cancer-associated hypercalcemia.

Type of model	Model Name and Tumor Origin	Strain	References
Allogenic	HSDM1 fibrosarcoma	Swiss albino mice	[119]
	CE mammary carcinoma	BALB/cfC3H	[120,121]
	5T2MM and 5T33MM multiple myeloma	C57BL/KalwRiJ	[122,123]
Transgenic	Tax+ lymphoma/leukemia	Tax+ C57B6/SJL	[124]
Infusion	PTHrP, IL-1, IL-11, TNF-α	-	[50,125,126]
Induction	Cutaneous administration of dimethylbenzanthracene induced squamous cell carcinoma	CD1 mice	[50,118]
Xenograft	Lung cancer	Nude mice	[127,128,129]
	Squamous cell carcinoma		[128,130]
	Renal cell carcinoma		[131,132]
	Urothelial cancer		[133]
	Esophageal carcinoma		[134,135]
	Melanoma		[136,137]
	Mammary cancer		[138]
	Pancreatic cancer		[139]
	Colon cancer		[140,141]
	Ovarian cancer		[142]
	Adult T-cell leukemia/lymphoma	SCID, NOD/scid	[143,144,145]
	Multiple myeloma		[146,147]
	Ovarian cancer		[148]

**Table 4:** Rat models of cancer-associated hypercalcemia.

Type of Model	Model Name	Tumor	Strain	References
Allogenic	Rice-500	Leydig cell	Fisher rat	[169,170,171]
	Walker 256	Mammary gland carcinoma	Fisher rat	[181,182,183]
	IP	Pulmonary carcinoma	Fisher rat	[189]
Infusion	PTHrP	-	-	[126,168]
Induction	Nitrosourea compounds	Mammary gland carcinoma	Sprague-Dawley	[192]
Xenograft	Uterine carcinoma	Uterine carcinoma	Human	[190,192,195]
Xenograft	OMC-1	Fibrosarcoma	Human	[193]
